# Dimensionality reduction-based fusion approaches for imaging and non-imaging biomedical data: concepts, workflow, and use-cases

**DOI:** 10.1186/s12880-016-0172-6

**Published:** 2017-01-05

**Authors:** Satish E. Viswanath, Pallavi Tiwari, George Lee, Anant Madabhushi, for the Alzheimer’s Disease Neuroimaging Initiative

**Affiliations:** Department of Biomedical Engineering, Case Western Reserve University, 10900 Euclid Ave, Wickenden 523, Cleveland, OH USA

**Keywords:** Data fusion, Imaging, Non-imaging, Kernels, Dimensionality reduction

## Abstract

**Background:**

With a wide array of multi-modal, multi-protocol, and multi-scale biomedical data being routinely acquired for disease characterization, there is a pressing need for quantitative tools to combine these varied channels of information. The goal of these integrated predictors is to combine these varied sources of information, while improving on the predictive ability of any individual modality. A number of application-specific data fusion methods have been previously proposed in the literature which have attempted to reconcile the differences in dimensionalities and length scales across different modalities. Our objective in this paper was to help identify metholodological choices that need to be made in order to build a data fusion technique, as it is not always clear which strategy is optimal for a particular problem. As a comprehensive review of all possible data fusion methods was outside the scope of this paper, we have focused on fusion approaches that employ dimensionality reduction (DR).

**Methods:**

In this work, we quantitatively evaluate 4 non-overlapping existing instantiations of DR-based data fusion, within 3 different biomedical applications comprising over 100 studies. These instantiations utilized different knowledge representation and knowledge fusion methods, allowing us to examine the interplay of these modules in the context of data fusion. The use cases considered in this work involve the integration of (a) radiomics features from T2w MRI with peak area features from MR spectroscopy for identification of prostate cancer in vivo, (b) histomorphometric features (quantitative features extracted from histopathology) with protein mass spectrometry features for predicting 5 year biochemical recurrence in prostate cancer patients, and (c) volumetric measurements on T1w MRI with protein expression features to discriminate between patients with and without Alzheimers’ Disease.

**Results and conclusions:**

Our preliminary results in these specific use cases indicated that the use of kernel representations in conjunction with DR-based fusion may be most effective, as a weighted multi-kernel-based DR approach resulted in the highest area under the ROC curve of over 0.8. By contrast non-optimized DR-based representation and fusion methods yielded the worst predictive performance across all 3 applications. Our results suggest that when the individual modalities demonstrate relatively poor discriminability, many of the data fusion methods may not yield accurate, discriminatory representations either. In summary, to outperform the predictive ability of individual modalities, methodological choices for data fusion must explicitly account for the sparsity of and noise in the feature space.

## Background

Predictive, preventive, and personalized medicine has the potential to transform clinical practice by enabling the use of multi-scale, multi-modal, heterogeneous data to better determine the probability of an individual contracting certain diseases and/or responding to a specific treatment regimen. These heterogeneous modalities may characterize either imaging (such as Magnetic Resonance Imaging (MRI), ultrasound, histology specimens) or non-imaging (gene-, protein-expression, spectroscopy) data, based on the method and type of data being acquired.

**Fig. 1:**
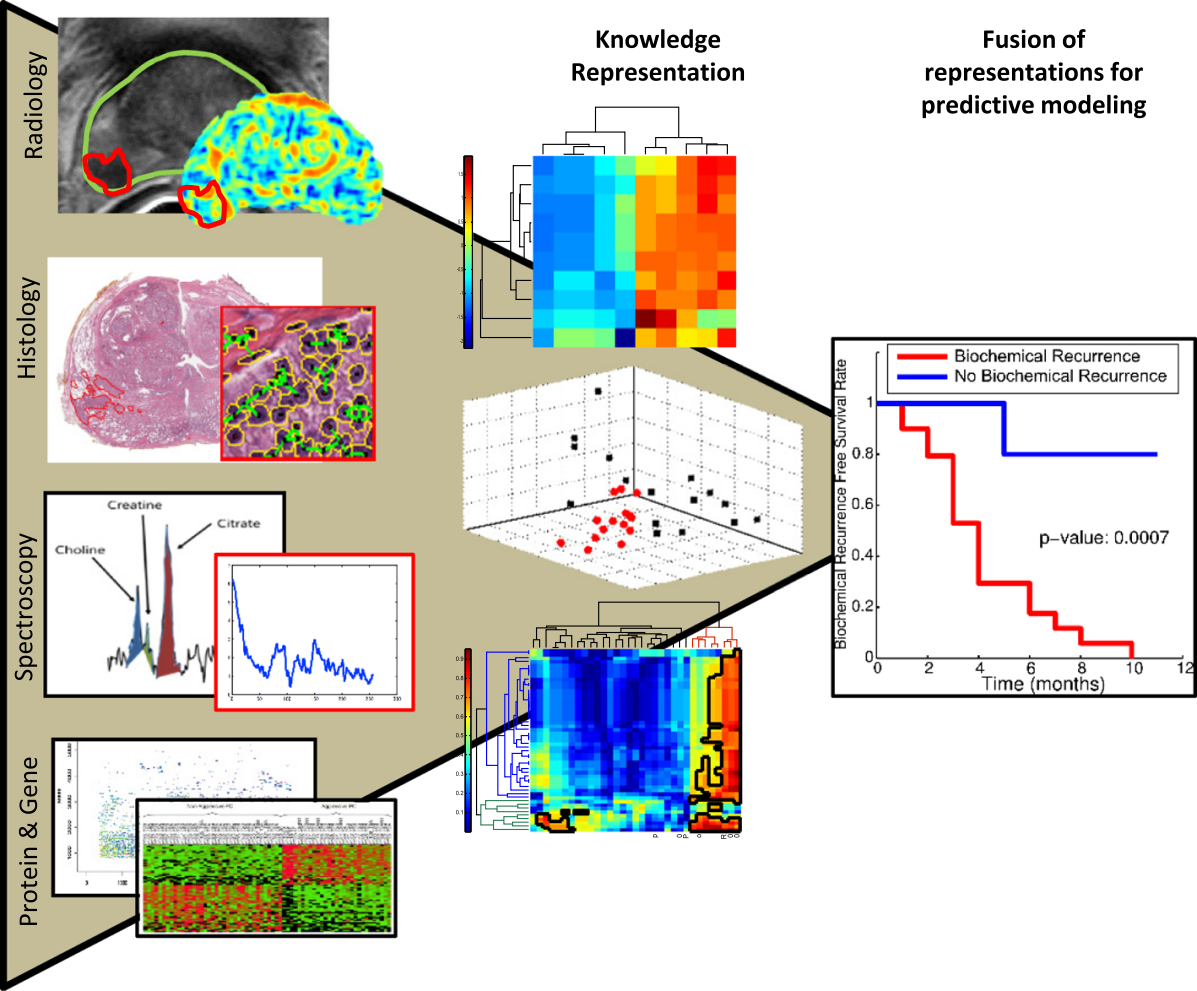
Illustration of data acquired at different length scales from imaging (radiology, pathology) and non-imaging (MR spectroscopy, protein expression) data, which could be combined to create fused predictors of disease aggressiveness and treatment outcome. In this illustration we use the example of prostate to illustrate the types of data that might be acquired before and after radical prostatectomy. In vivo information acquired prior to prostatectomy includes MR imaging and spectroscopy, while the surgical specimen yields digitized histological sections as well as undergoing genomic profiling via mass spectrometry. The middle column of the illustration depicts different knowledge representation methods (e.g. dimensionality reduction, co-association matrices) for uniformly representing multi-modal data. Once represented in a common space, these features can be combined to create a predictive model. An application of this predictive model could include survival curve analysis (far right column, obtained by combining histologic and proteomic features) for identification of prostate cancer patients who will later suffer from biochemical recurrence within 5 years (*red*) from those who will not (*blue*)

These modalities also have differing dimensionalities, where MRI, ultrasound are scalar intensity values, while spectroscopy is a multi-dimensional signal comprising metabolite concentrations at every image voxel (Fig. 1). More crucially, each of these modalities capture different types of information about the disease at different length scales. For example, gene expression levels represent cellular scale observations; changes in which would result in a phenotypic structural or vascular difference on tumor morphology that is captured at the pathologic scale via standard H&E tissue specimens [1]. While data acquired at different length scales may be considered to capture complementary characteristics (structural versus biological), the associated information is represented via fundamentally different data types (images versus molecular concentrations).

We define *multi-modal data fusion* as the process of combining a variety of complementary measurements from different data modalities, existing at different length scales, into an integrated predictor [1]. Combining complementary sources of information in this manner to yield a more comprehensive characterization of a disease or tissue region has been demonstrated to yield a more accurate predictor than using any individual data modality [2, 3].

Recently, our group and several others have explored different dimensionality reduction (DR) based fusion approaches, such as linear or non-linear projections [4–7], multi-kernel learning [8, 9] or feature selection [10–12] to address the challenge of multi-modal data fusion; specifically involving imaging and non-imaging data modalities. Note that while there is a plethora of fusion methodologies, we choose to focus here on DR-based multimodal data fusion.

Consider the publicly available ADNI database which contains imaging (MRI and PET), as well as non-imaging (genetics, cognitive tests, CSF and blood biomarkers) information for a population of patients with and without Alzheimer’s disease. Using the ADNI database, multiple data fusion methodologies have been proposed to integrate these different data types to build a fused predictor for Alzheimer’s disease, including classifier-based [13], dimensionality reduction-based [7], as well as multi-kernel learning-based [14]. Given that these methods all attempt multi-modal data fusion, one can posit the following questions:

- How are these approaches similar or different from one another?
- How does a particular method compare to other fusion methods applied to same dataset, either methodologically or in terms of performance?
- How can a particular method be selected over any other for a new application i.e. do the methods generalize or do they require specific types of information?

Motivated by the seminal work by Yan et al. [15], who demonstrated that different dimensionality reduction methods can be formulated as instantiations of the “generalized graph embedding” approach, in this paper we propose to identify common methods and thus an underlying workflow which govern existing multi-modal data fusion strategies. Further, we will compare a subset of these data fusion methods to better understand the contributions of the individual modules that comprise a data fusion strategy.

The rest of the paper is organized as follows. We first briefly define the specific steps (*representation* and *fusion*) typically followed within a multi-modal data fusion strategy, based on a summary of existing work in this domain. We then provide a detailed description of the different modules that have been previously utilized for data representation as well as data fusion. Experiments to demonstrate the application of multi-modal data fusion in the context of different diagnostic and prognostic clinical problems are then described, followed by the results of quantitative and qualitative evaluation of representative data fusion strategies within these applications. Note that while we have attempted to diversify in terms of our choice of datasets and methods employed, this work is not meant as a comprehensive evaluation of all possible imaging and non-imaging fusion methods and datasets. For instance, we have not extensively explored the popular canonical correlation class of fusion approaches [6]. We have instead opted to systematically compare and relate a few different representative multi-modal data fusion strategies in the context of different clinical applications, to provide a basic understanding of the interplay of different individual modules that can comprise a data fusion method. As all the techniques compared in this study involved projecting the data modalities to construct a reduced fused representation, we have essentially focused on DR-based multimodal data fusion. Finally, we conclude by summarizing our takeaways and directions for future work.

### Generalized overview of a dimensionality reduction-based multi-modal data fusion strategy

Table 1 summarizes a number of recently presented methods for multi-modal data fusion, including the variety of data that has been examined and the different methods that have been utilized in each case. Based on the literature, we observe that there appear to be two specific steps that are utilized (either explicitly or implicitly):

**Table 1:** Brief review of multi-modal data fusion methods from the literature and methodologies that have been used

Reference	Data	Method
Moutselos et al. [65]	Skin images	Combining features into a confusion matrix
	Gene expression	
Golugula et al. [6]	Histopathology	Correlating features via CCA, combining CCA-based confusion matrices
	Proteomics	
Dai et al. [20]	sMRI	Construct classifiers from features, weighted combination of classifier decisions
	fMRI	
Gode et al. [66]	mRNA	Compute LDR/classifier decisions, unweighted combination of LDR- or classifier-based confusion matrices
	miRNA	
Raza et al. [22]	Gene-expression	Compute classifier decisions, unweighted combination of classifier decisions
	FNAC	
Sui et al. [67]	DTI	Correlate features via CCA, unweighted combination of CCA-based confusion matrices
	fMRI	
Wolz et al. [7]	T1-w MRI	Compute LDR, weighted combination of LDR-based confusion matrices
	ApoE genotype, A *β*_1−42_	
Wang et al. [62]	T1-w MRI, FDG-PET	Feature selection, weighted concatenation of selected features
	Gene-expression	
Lanckriet et al. [9]	Protein expression	Compute kernel representations, weighted combination of kernels
	Gene-expression	
Yu et al. [68]	Text ontologies	Compute kernel representations, fuse kernel-based confusion matrices
	Gene-expression	
Higgs et al. [54]	CT	Compute LDR, fuse LDR maintaining manifold structure
	Gene-expression	
Lee et al. [4]	Gene-expression	Compute LDR, unweighted concatenation of LDR
	Histopathology	
Viswanath et al. [5]	T2-w	Compute LDR, combine LDR-based confusion matrices using label information
	ADC, DCE	
Tiwari *et al* [8]	T2-w MRI	Compute kernel representations, weighted LDR-based combination of kernels using label information
	MRS	

*CCA* Canonical Correlation Analysis, *LDR* Low-Dimensional Representation. See Description of methods utilized for multi-modal data fusion section for more details

1. *Knowledge representation*: We define this as transforming the individual data modalities into a common space where modality differences in terms of scale and dimensionality are removed. This includes methods such as kernel representations [16], low-dimensional representations (LDR) [17], or classifier-based decisions [18].
2. *Knowledge fusion*: We define this as combining multiple different knowledge representations into a single integrated result to build a fused predictor, such that complementary information from different modalities is leveraged as best possible. Methods utilized in this regard include confusion matrices, weighted or unweighted combinations, as well as concatentation.

**Fig. 2:**
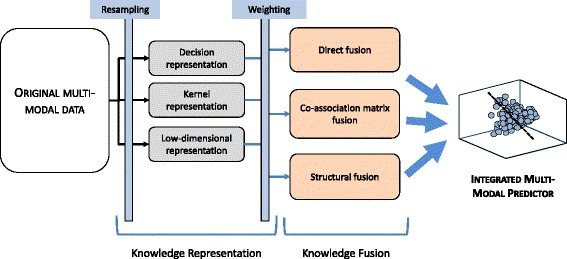
Generalized overview of steps followed for DR-based multimodal data fusion. Knowledge representation refers to transforming each modality individually into a space where modality-specific scale and dimensionality differences are removed. Resampling allows for generation of multiple representations from each data modality to try and maximize the information extracted from it. Knowledge fusion then combines different representations into a single integrated result to build a fused predictor. Weighting enables building of a fused result where the data modalities are differentially considered depending on how well they individually characterize the data. The final fused result is expected to leverage the complementary information from different modalities as best as possible

Based on our summary of the literature in Table 1, we further conceptualize the interplay of these two steps in the context of multi-modal data fusion as illustrated in Fig. 2. We have additionally incorporated commonly used strategies of resampling (generating multiple representations from each data modality) as well as weighting (differentially considering data modalities depending on their contributions) into this series of steps. The different options for representation as well as fusion have been enumerated in the flowchart; note that any fusion method could be used with any representation method. This indicates that a wide variety of data fusion strategies can be enumerated, however, we must once again note that the current study is not intended as a comprehensive review of all these possible methods. The representative strategies we have chosen to compare in the current study have instead been chosen based on combining different aspects of the workflow depicted in Fig. 2, and all of them involve some form of dimensionality reduction.

## Methods

### Description of methods utilized for multi-modal data fusion

### Notation

We define the original feature space associated with samples *c*_*i*_ and *c*_*j*_ for modality *m* as $\mathcal {F}_{m} = \left [\mathbf {F}_{m}(c_{1}),\dots,\mathbf {F}_{m}(c_{N})\right ]$, *i,j*∈{1,…,*N*}, *m*∈{1,…,*M*}, where *N* is the number of samples and *M* is the number of modalities. The corresponding class label for sample *c*_*i*_ is given as *ω*_*i*_∈[0,1].

### Knowledge representation

The primary goal of this step is to transform different multi-modal data channels into a common space to overcome inherent dimensionality and scale differences. Representation facilitates subsequent data fusion step by (a) preserving information from each of the input heterogeneous data modalities, while (b) accounting for factors that would be detrimental to combining this information.

**Decision representations** This class of approaches involve deriving classifier outputs from independent data channels [18]. For example, Jesneck et al. [19] calculated individual sets of probabilities from different imaging modalities (mammograms, sonograms) as well as patient history (non-imaging). These sets of classifier probabilities were then quantitatively fused to yield an integrated classifier for improved breast cancer diagnosis (as the modalities had been transformed into a common classifier probability space).

For each modality *m*∈*M*, decision representation involves calculating a probability for each sample as belonging to the target class, denoted as *h*_*m*_(*c*_1_)…,*h*_*m*_(*c*_*N*_), 0≤*h*_*m*_≤1, which may be done via a wide variety of classifier methods that exist [18]. While classifier-based approaches have seen extensive use in as an implicit form of data fusion [20–22], one of the major disadvantages to this class of approaches is that all inter-source dependencies between modalities are lost, as each modality is being treated independently when computing the decision representation [4].

**Kernel representations** Kernels are positive definite functions which transform the input data to an implicit dot product similarity space [16], and in typical use, different kernels are used to represent each data modality [9], with the advantage being the flexibility to tweak and fine-tune the kernel depending on the type of data being considered [23].

For each modality *m*∈*M*, the kernel representation is calculated as *K*_*m*_(*c*_*i*_,*c*_*j*_)=〈*Φ*(**F**_*m*_(*c*_*i*_),*Φ*(**F**_*m*_(*c*_*j*_))〉, where *Φ* is the implicit pairwise embedding between the feature vectors **F**_*m*_(*c*_*i*_) and **F**_*m*_(*c*_*j*_) being calculated between every pair of points *c*_*i*_ and *c*_*j*_, *i,j*,∈{1,…,*M*}, for modality *m*, while 〈.〉 denotes the dot product operation.

Kernels and multi-kernel learning are one of the most powerful representation strategies which has found wide application in many different domains [14,24–26]. However, in addition to being computationally expensive, there is a lack of transparency in relating kernel representations to the input multi-modal data, as it is not possible to create an interpretable visualization of the joint kernel space.

**Low-dimensional representations (LDR)** Dimensionality reduction transforms input data to a low-dimensional space while preserving pairwise relationships between samples as best possible [17]. Typically, these pairwise relationships can be quantified via affinities or distances (as used by methods such as spectral embedding [27]); however, it is also possible to utilize measures such as covariance as considered within canonical correlation analysis (CCA) [6,28] or principal component analysis (PCA) [29].

Low-dimensional representations first require calculation of an *N*×*N* confusion matrix *W*=[*w*_*ij*_] which attempts to capture pairwise relationships between objects *c*_*i*_ and *c*_*j*_, *i,j*∈{1,…,*N*}, *N* being the total number of samples. The corresponding low-dimensional representation ***y*** can be obtained via Eigenvalue decomposition as,

1 $$  W\boldsymbol{y} = \lambda \mathcal{D}\boldsymbol{y},  $$

with the constraint $\boldsymbol {y}^{T}\mathcal {D}\boldsymbol {y}=1$, where $\mathcal {D}_{ii}=\sum _{j}{w_{ij}}$. Given *M* modalities, *W*_*m*_ is calculated for every *m*∈*M*, each of which are then subjected to Eigenvalue decomposition to yield the low-dimensional representations ***y***_*m*_. Low-dimensional representations have proven very popular for biomedical applications [30–33], especially as they enable informative visualizations (such as cluster plots) while ensuring computational tractability. Similar to kernel representations, depending on the LDR method used, one cannot always relate the low-dimensional representation to the original multi-modal data.

### Generation of multiple representations (resampling)

The robustness and generalizability of representation techniques has been shown to improve when multiple representations of input data are generated and combined [5,34–36]. For example, combining multiple classifier outputs into an “ensemble” classifier result has been demonstrated to yield better classification accuracy and generalizability than any individual classifier (both analytically and empirically) [34,37]. This idea of calculating a number of representations is typically implemented by resampling a given dataset as demonstrated for classifier decisions [34], projections [38], and clusterings [39].

Thus, rather than calculate a single representation per modality (i.e. generating *M* representations for *M* distinct modalities), *n* “weak” representations could be generated for each of *M* modalities, in total yielding *nM* representations of heterogeneous data modalities.

These may be generated in any of the following ways:

- *Perturbing the samples*: Given a set of *N* samples in a set *C*, *n* bootstrapped sets *C*_1_,*C*_2_,…,*C*_*n*_⊂*C* (with replication) are created, which in turn will yield *n* different representations. Each of *C*_1_,*C*_2_,…,*C*_*n*_ will consist of samples drawn at random from *C*, but with replacement, such that every sample *c*∈*C* may be repeated multiple times across all of *C*_1_,*C*_2_,…,*C*_*n*_. This approach has been termed “bootstrapped aggregation” (or bagging [37]).
- *Perturbing the parameters*: All knowledge representation schemes (kernels, decisions, low-dimensional) are known to be sensitive to the choice of parameters used [40–42]. For example, a neighborhood parameter must be optimized for calculating an accurate low-dimensional representation via locally linear embedding [42,43] or for constructing an accurate *k*-nearest neighbor classifier model [44]. A range of *n* possible parameter values can be used to generate *n* different “weak” representations [45].
- *Perturbing the features*: Similar to perturbing the samples, we can create *n* bootstrapped sets of features with replication.
  By varying the feature space input to the representation scheme, it is possible to generate *n* distinct “weak” representations [5].

### Knowledge fusion

Given *nM* knowledge representations of *M* input heterogeneous modalities, the objective of *knowledge fusion* [9,19,34] is to combine multiple different representations into a single integrated result, denoted as $\widehat {\mathcal {R}}$. Note that this fusion may involve combining the knowledge representations directly (i.e. combining kernels or low dimensional representations) or by preserving specific relationships associated with a representation technique (e.g. affinity-based or structure-based fusion). $\widehat {\mathcal {R}}$ will be subsequently utilized to build a comprehensive predictor for a given dataset [23,46,47].

**Direct fusion** The most popular class of fusion strategies involve *directly combining* a set of knowledge representations either through simple concatenation or a weighted combination. Concatenation has most popularly been used for combining information extracted from multiple imaging modalities which are in spatial alignment [2,48–50] i.e. intensity values from across multiple modalities are concatenated at every spatial location into a single feature vector.

Calculating a final fused representation, $\widehat {\mathcal {R}}$, based on a set of representations *ϕ*_*t*_,*t*∈{1,…,*nM*}, can be written as, *ϕ*∈{**F**,*h*,***y***,*K*},

2 $$  \widehat{\mathcal{R}}={\underset{{\forall t}}{\xi}}[\phi_{t}],  $$

where *ξ* may be a weighted or unweighted combination function (including concatenation). For example, $\widehat {\mathcal {R}}=\sum _{\forall t}\alpha _{t}[h_{t}]$ corresponds to a weighted combination of decision representations (*α* corresponds to the weight), as typically performed via Adaboost [51]. Similarly, the combination method adopted in [46], where PCA-based representations of MRI (denoted as ***y***_*MRI*_) and MR spectrocopy (denoted by ***y***_*MRS*_) were concatenated into a unified predictor, can be rewritten as $\widehat {\mathcal {R}}=[\boldsymbol {y}_{MRI}, \boldsymbol {y}_{MRS}]$.

**Co-association matrix fusion** This fusion approach involves integrating information *being derived from* the knowledge representations (i.e. properties of the representations are extracted and combined). This information is captured within what we term a *co-association matrix*, which is then decomposed to yield a single, unified representation. Typically Eigenvalue decomposition is utilized for the latter as it will yield a mathematically interpretable representation of an input square matrix.

We denote the co-association matrix as $\mathcal {W}_{t} = \delta (\phi _{t})$, where *δ* is any function used to quantify the information within the representations *ϕ*_*t*_, *t*∈{1,…,*nM*}, *ϕ*∈{**F**,*h*,***y***,*K*}. These $\mathcal {W}_{t}, t\in \{1,\dots,nM\}$, can then be combined as $\widehat {\mathcal {W}} = \xi _{\forall t}[\mathcal {W}_{t}]$, where *ξ* is a weighted or unweighted combination function. The final fused representation, $\widehat {\mathcal {R}}$, may then be calculated via Eigenvalue decomposition as,

3 $$  \widehat{\mathcal{W}}\widehat{\mathcal{R}} = \Lambda \widehat{\mathcal{D}} \widehat{\mathcal{R}},  $$

such that $\widehat {\mathcal {R}}^{T}\widehat {\mathcal {D}}\widehat {\mathcal {R}}=1$ and $\widehat {\mathcal {D}}_{ii}=\sum _{j}{\widehat {\mathcal {W}}_{ij}}$ (similar to Eq. 1).

Note that depending on the type of association being captured in $\mathcal {W}_{t}$ and the type of representation *ϕ*_*t*_, some modifications to Eq. 3 may be required to obtain an appropriate $\widehat {\mathcal {R}}$. For example, when considering kernel representations, Eq. 3 is modified to result in a multi-kernel Eigenvalue decomposition problem as follows,

4 $$  \widehat{\mathcal{K}}\widehat{\mathcal{W}}\widehat{\mathcal{K}}^{T}\widehat{\mathcal{R}} = \Lambda \widehat{\mathcal{K}}\widehat{\mathcal{D}}\widehat{\mathcal{K}}^{T} \widehat{\mathcal{R}},  $$

subject to same conditions as for Eq. 3. Here, $\widehat {\mathcal {K}}=\xi _{\forall t}[K_{t}]$, *t*∈{1,…,*nM*}, is the combined kernel representation based on the combination function *ξ*.

Co-association matrix fusion can be seen to encompass a wide variety of previous work, including combining pairwise distances extracted from multiple low-dimensional representations [5,45], combining correlations extracted from multiple kernel representations [52], or combining CCA-based representations via regularization [6].

**Structural fusion** Fusing the structure inherent to a knowledge representation [53,54] is a perhaps lesser explored approach to data fusion. In one of its earliest applications, Higgs et al. [54] demonstrated that spectral embedding revealed implicit complementary manifold structure information in both image and microarray data, which could be useful in classification. The idea of fusing representations (derived from different data modalities) at a structural level has thus been primarily explored in the context of low-dimensional representations [53,55,56].

Given a set of representations *ϕ*_*t*_, *t*∈{1,…,*nM*}, *ϕ*∈{**F**,*h*,***y***,*K*}, structural fusion first involves some form of “representation alignment” to ensure that all of *ϕ*_*t*_ lie in the same co-ordinate frame-of-reference, i.e., calculating $\widehat {\phi }_{t} = \mathrm {T}(\phi _{t})$, where T denotes the transformation required to align the representation into a unified frame-of-reference. For example, point correspondences have been used to drive an alignment of low-dimensional representations to one another, in previous work [57].

Once aligned, the final fused representation, $\widehat {\mathcal {R}}$, could be obtained via,

5 $$  \widehat{\mathcal{R}} = {\underset{{\forall t}}{\xi}}(\widehat{\phi}_{t}),  $$

where *ξ* denotes the combination function. In addition to applications demonstrated in learning [55] and retrieval [56], structural fusion was utilized by Sparks et al. [35] to develop a parametrized shape model to combine information from across multiple aligned low-dimensional representations and thus distinguish between tumor sub-types via pathology data.

### Weighted and unweighted data fusion

For each of the data fusion strategies above, the combination function *ξ* enables either a weighted or an unweighted combination of the different data modalities. Calculation of weights requires quantification of the relative contributions of the individual data modalities, and ensures that the resulting unified representation accurately captures these contributions. Further, the unified representation that leverages weighting may be expected to demonstrate better class separability compared to a naive, unweighted combination (or concatenation) of these data modalities, as demonstrated in the context of decision and low-dimensional representations. However, learning optimal weights for the data modality typically requires some form of class information.

In previous work, decision representations have been extensively explored in terms of both unweighted [37] and weighted [51] combinations, while kernel representations have classically been considered within weighted multi-kernel formulations [16,23] alone. By contrast, low-dimensional representations have typically been combined within an unweighted formulation [5,45]. Label information has, however, been used to regularize low-dimensional representations [6,7] (i.e. to minimize outliers and ensure a smooth continuum between different classes). Thus, for each of the knowledge fusion strategies above, it is possible to utilize either of the following:

- *Unweighted*: Instead of using label information, a data-driven estimation is typically utilized. For example, both Tiwari et al. [45] and Viswanath et al. [5] utilize the median as a maximum likelihood estimator across multiple co-association matrices $\mathcal {W}_{t},t\in \{1,\dots,nM\}$, (derived from corresponding low-dimensional representations ***y***_*t*_). This results in a unified $\widehat {\mathcal {W}}$, which then undergoes Eigenvalue decomposition as detailed in Eq. 3.
- *Weighted*: An optimization function is utilized to calculate different weights for each input representations. In the presence of labeled information, this function could optimize classification accuracy; alternate objective functions could include an unsupervised clustering measure or a similarity measure. For example, label information has been used both for multi-kernel learning [58] as well as for constructing a semi-supervised representation [8].

### Experimental design

To better understand the interplay and contributions of different modules that can be utilized for multimodal data fusion, we have implemented four representative DR-based data fusion methods and evaluated their performance in three distinct clinically relevant problems. Table 2 summarizes the 3 problems and different types of data considered in this work. Note that each clinical problem was identified such that it involves heterogeneous data integration of different data types and dimensionalities, including fusion of radiology and gene-expression data (radio-genomics), MR imaging and spectroscopy data (radio-omics), and histology and protein-expression data (histo-omics). Further, for each clinical problem, the features being extracted are at different length scales (per-location, per-region, and per-patient basis), resulting in different ratios for the number of samples (*N*) to the number of features (*P*). Note that while we have attempted to diversify in terms of our choice of datasets and methods employed, this work is not meant as a comprehensive evaluation of all possible imaging and non-imaging fusion methods and datasets.

**Table 2:** Summary of the three clinical problems and data cohorts utilized to evaluate the GFA

Dataset	# Studies	Modalities	Clinical problem addressed
*S*_1_	77	T1-w MRI, protein-expression	Differentiating Alzheimer’s patients from normal subjects
*S*_2_	40	Histology, protein expression profiles	Predicting biochemical recurrence in prostate cancer
*S*_3_	36 (3000 voxels)	T2-w MRI, MR spectroscopy	Detecting prostate cancer on a per-voxel basis

### Multimodal data fusion strategies compared

For each problem, four distinct multimodal data fusion methods were implemented, each of which utilized different combinations of fusion and representation modules (see Table 3 for details). In addition to having being previously published, these instantiations each utilize different representation and fusion methods, but with the common step of using dimensionality reduction to construct the final fused representation. These instantiations were then systematically compared to the individual imaging and non-imaging modalities in terms of their predictive accuracy for each of these problems. For the purposes of readability, we utilize the acronym DFS (Data Fusion Strategy) in Table 3.

**Table 3:** Summary of different DR-based multimodal data fusion methods considered in this work

Strategy	Resampling	Representation	Weighting	Fusion
DFS-DD	-	Decision	Unweighted	Direct fusion (AND operation)
DFS-EC	Feature perturbation	PCA	Unweighted	Co-association matrix fusion
DFS-KC	-	Kernels	Weighted, semi-supervised	Co-association matrix fusion
DFS-ES	-	LLE	Unweighted	Structural fusion

*DFS* Data Fusion Strategy, *DD* Decision representation, Direct fusion, *EC* Embedding representation, Co-Association fusion, *KC* Kernel representation, Co-Association fusion, *ES* Embedding representation, Structural fusion

### Dataset *S*_1_: MRI, proteomics for Alzheimer’s disease identification

*S*_1_ requires the construction of a classifier to differentiate Alzheimer’s Disease (AD) patients from a normal population, based on quantitatively integrating area and volume measurements derived from structural T1-weighted brain MRI with corresponding plasma proteomic biomarkers.

A total of 77 patients were identified from the Alzheimer’s Disease Neuroimaging Initiative (ADNI) database (adni.loni.usc.edu), of which 52 had been catalogued as having AD while the remainder were normal healthy controls. The ADNI was launched in 2003 as a public-private partnership, led by Principal Investigator Michael W. Weiner, MD. The primary goal of ADNI has been to test whether serial magnetic resonance imaging (MRI), positron emission tomography (PET), other biological markers, and clinical and neuropsychological assessment can be combined to measure the progression of mild cognitive impairment (MCI) and early Alzheimer’s disease (AD). For up-to-date information, see www.adni-info.org. Patients were included in *S*_1_ based on having both (a) a structural 1.5 T T1w MRI scan acquired per the standardized ADNI protocol, and (b) plasma proteomics data, for which detailed collection and transportation protocols are described on the ADNI website (http://www.adni-info.org/Scientists/ADNIScientistsHome.aspx). The brain regions known to be most affected by AD had been segmented and quantified via the widely used FreeSurfer software package (http://surfer.nmr.mgh.harvard.edu/), that was run on each T1w MRI scan, yielding a total of 327 features (that were available for download). Similarly, plasma proteomics had been extracted through a multiplex immunoassay panel of blood samples to yield a protein expression vector (that was available for download). These features are summarized in the Appendix (Table 5), and described in more detail on the ADNI webpage (http://adni.loni.ucla.edu/).

### Dataset *S*_2_: Histology, proteomics for prostate cancer prognosis

*S*_2_ requires building a prognostic classifier that can distinguish between prostate cancer (CaP) patients that are at risk for disease recurrence versus those who are not, using pathology and proteomic information acquired immediately after radical surgery.

A cohort of 40 CaP patients was identified at the Hospital at the University of Pennsylvania, all of whom underwent radical prostatectomy. Half of these patients had biochemical recurrence following surgery (within 5 years) while the other half did not. For each patient, a representative histological prostatectomy section was chosen and the tumor nodule identified. Mass spectrometry was performed at this site to yield a protein expression vector. The resulting 650 dimensional proteomic feature vector consisted of quantifiable proteins found across at least 50% of the studies. A corresponding set of 189 histology features were extracted based on using quantitative histomorphometry on the digitized slide specimen and included information relating to gland morphology, architecture, and co-occurring gland tensors. Both sets of features are summarized in the Appendix (Table 6), and have been described in more detail in Lee et al. [11].

### Dataset *S*_3_: Multiparametric MRI for prostate cancer detection

*S*_3_ requires quantitatively combining 2 different MRI protocols for accurately identifying locations of prostate cancer (CaP) in vivo, on a per-voxel basis: (a) T2-weighted MRI reflecting structural imaging information about the prostate, where every location is characterized via a scalar image intensity value, and (b) MR spectroscopy data which captures the concentrations of specific metabolites in the prostate, and every location is represented as a vector or spectrum.

A total of 36 1.5 Tesla T2w MRI, MRS studies were obtained prior to radical prostatectomy from University of California, San Francisco. These patients were selected as having biopsy proven CaP, after which an MRI scan (including T2w MRI and MRS protocols) had been acquired. For every patient dataset, expert labeled cancer and benign regions (annotated on a per voxel basis) were considered to form the CaP ground truth extent, yielding a total of 3000 voxels. For each voxel, 6 MRS features were calculated based on calculating areas under specific peaks to determine deviations from predefined normal ranges [46]. 58 voxel-wise MRI features were extracted for quantitatively modeling image appearance and texture to identify known visual characteristics of CaP presence [46]. The specific features utilized are summarized in the Appendix (Table 7), and were extracted as described in Tiwari et al. [8].

### Evaluation measures

In order to compare the performance of different multimodal data fusion methods against each other, as well as against using the individual modalities, we formulated each of *S*_1_,*S*_2_,*S*_3_ as a 2-class classification problems. Classifier performance in segregating the two classes was used to quantify how well each of these strategies preserves information relevant to building such a predictor. Thus the parameters governing the creation of the integrated representation as well as for constructing the classifier were kept as consistent as possible for all 3 datasets.

**Classifier construction and evaluation** The Random Forests (RF) classifier [37] was utilized to construct classifiers in all experiments. RF uses the majority voting rule for class assignment by combining decisions from an ensemble of bagged (bootstrapped aggregated) decision trees.

The primary motivation for using RF over other classifier schemes were, (1) ability to seamlessly integrate a large number of input variables, (2) robustness to noise in the data, and (3) relatively few parameters that require tuning [59].

The RF implementation within MATLAB (*TreeBagger*) was utilized, where the number of bagged decision trees was set to 100, and each decision tree was generated through subsampling 66% of the input training feature space. A separate RF classifier was trained and evaluated on each of the 4 multimodal fusion methods (see Table 3) as well as on each the 2 individual data modalities (i.e. a total of 6 classifiers). Evaluation of the RF classifier in each case was done through ROC analysis, to yield an area under the receiver-operating characteristic curve (AUC) as a measure of performance for each method.

Classifier robustness was determined via a randomized three-fold cross validation procedure, with segregation of data on a per-patient basis. Each run of three-fold cross validation involved randomly dividing a given dataset into three folds, following which 2 folds (i.e. 2/3 ^*rd*^) were used for training and the remaining fold (1/3 ^*rd*^) for testing. This is repeated until all the samples are classified within each dataset. This randomized cross-validation was then repeated a total of 25 times, and done separately for each of the 6 RF classifiers.

**Statistical testing** Through the three-fold cross-validation procedure, each classifier yielded a set of 25 AUC values (corresponding to each cycle of the procedure) and for each of 6 strategies being compared. Multiple comparison testing to determine statistically significant differences in AUC values for each dataset considered (i.e. within the results for each of *S*_1_, *S*_2_, *S*_3_) was performed using the Kruskal–Wallis (K-W) one-way analysis of variance (ANOVA) [60]. The K-W ANOVA is a non-parametric alternative to the standard ANOVA test which does not assume normality of the distributions when testing. The null hypothesis for the K-W ANOVA was that the populations from which the AUC values originate have the same median. Based off the results of a K-W ANOVA, multiple comparison testing was performed to determine which representations showed significant differences in performance in a given problem.

## Results

Table 4 summarizes the mean as well as the standard deviation in AUC values for each of 6 strategies, in each of the 3 classification tasks considered (calculated over 25 runs of three-fold cross validation). The highest performing strategy in each task is highlighted in bold.

**Table 4:** Mean and standard deviation in AUC values (obtained via three-fold cross validation) for datasets *S*_1_, *S*_2_, and *S*_3_, while utilizing different DR-based multimodal data fusion methods (see Table 3 for details)

Strategy	Dataset *S*_1_	Dataset *S*_2_	Dataset *S*_3_
Non-imaging	0.774 ± 0.043	0.511 ± 0.078	0.771 ± 0.009
Imaging	0.885 ± 0.034	0.503 ± 0.076	0.564 ± 0.036
DFS-DD	**0.905 ± 0.035**	0.496 ± 0.079	0.752 ± 0.026
DFS-EC	0.675 ± 0.065^a^	0.465 ± 0.111	0.720 ± 0.020
DFS-KC	0.888 ± 0.040	**0.808 ± 0.067** ^b^	**0.857 ± 0.009** ^b^
DFS-ES	0.789 ± 0.035	0.531 ± 0.086	0.748 ± 0.013

For baseline performance comparison, AUC values for the individual data modalities are also reported

^a^indicates that the result was statistically significantly worse than comparative strategies

^b^indicates that the result was statistically significantly better than comparative strategies

The best performing data fusion strategy for each classification task is highlighted in bold

### Experiment 1: Integrating MRI and proteomics to identify patients with Alzheimer’s disease

DFS-DD (decision representation, direct fusion) demonstrated the highest overall AUC value, which can be directly attributed to the relatively high performance of the individual protocols (AUC of 0.77 for non-imaging, 0.88 for imaging data). However, the 3 top performing strategies (DFS-DD, DFS-KC, imaging data) also did not demonstrate any statistically significant differences in their performance in a Kruskal-Wallis test, indicating they were all comparable in terms of predictive performance. The least successful method was DFS-EC (embedding representation, co-association fusion), which demonstrated statistically significantly worse classifier performance compared to all the remaining strategies.

These results imply that when the input features have relatively high discriminability, multimodal data fusion that utilizes a simple representation (decisions) and a simple fusion (direct) approach, as utilized by DFS-DD, can be highly effective for creating an accurate predictor.

### Experiment 2: Integrating histopathology and proteomics to predict prostate cancer recurrence after surgery

DFS-KC (kernel representation, co-association fusion) yielded the highest AUC value in Dataset *S*_2_, and was also statistically significantly better than any alternative strategy (*p*=2.14*e* ^−12^). All the remaining strategies performed comparably, albeit relatively poorly (AUC ≈0.5), with no significant differences in their classifier performance. In comparison to dataset *S*_1_, it appears that dataset *S*_2_ has relatively poor features associated with the imaging and non-imaging modalities. As a result, most data fusion strategies (as well as the individual modalities) performed poorly for classification, possibly as they are unable to capture enough relevant information.

### Experiment 3: Integrating MRS and MRI to identify voxel-wise regions of prostate cancer recurrence after surgery in vivo

DFS-KC (kernel representation, co-association fusion) performed statistically significantly better than any alternative strategy in the classification task for Dataset *S*_3_ (*p*=9.81*e* ^−26^). Amongst the remaining strategies, DFS-DD and DFS-ES (embedding representation, structural fusion), as well as the non-imaging data, performed comparably and significantly better than DFS-EC or using imaging data alone. In this dataset, a mismatch can be observed in the relative discriminability of the individual modalities (AUC = 0.77 vs 0.56 for non-imaging vs imaging). Both kernel-based methods (DFS-KC) and embedding-based methods (DFS-ES) appear somewhat robust to this effect, however DFS-EC (embedding representation, co-association fusion) appears to have been affected by this issue. One possible factor contributing to the poor performance of DFS-EC may be the relatively low dimensionality of the MRS feature space (6 dimensions), which would prevent the resampling step of DFS-EC from being effective.

**Fig. 3:**
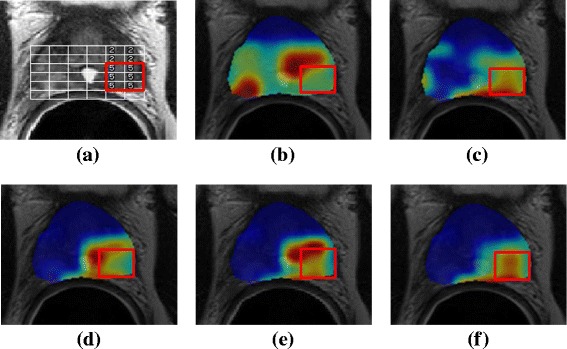
Sample predictive heatmaps for detection of prostate cancer in vivo through combining MRI and MRS data. **a** shows a T2w MRI section with the MRS grid overlaid in white. The expert annotation of cancer presence is also shown with a red outline around those voxels that were assessed as cancerous. Corresponding automated classification results are shown for using: **b** T2w MRI texture features alone, **c** MRS peak area metabolite ratios, **d** DFS-ES, **e** DFS-EC, **f** DFS-KC. These are visualized in the form of heatmaps, where red corresponds to higher probability of CaP presence. The expert annotation of CaP presence is also superposed via a red outline in each image

These conclusions are supported by the qualitative results in Fig. 3, which depicts representative classification results for detecting the presence of CaP on a voxel-wise basis in vivo via different strategies. These results were obtained by visualizing the voxel-wise RF classifier result for this section as a heatmap, where red corresponds to a higher likelihood of CaP presence. Classifying the MRS (Fig. 3 b) and T2w (Fig. 3 c) data modalities individually yields results that appear to detect CaP with widely varying accuracy (note poor overlap of red region with ground truth, depicted via a red outline). By contrast, multimodal data fusion via DFS-KC appears to show both optimal sensitivity and specificity, as much of the red in the heat map is located within the ground truth cancer region.

## Discussion

Our preliminary findings from this work were as follows,

- In terms of the knowledge representation module, a kernel-based method (DFS-KC) demonstrated the best classifier performance consistently across all 3 applications, implying that kernels may offer distinct advantages for multimodal data representation. This performance may have been further enhanced by the fact that DFS-KC utilized differential weighting for individual data modalities based on their contributions, in addition to using semi-supervised learning. However, we must note that this method was also amongst the most computationally expensive in terms of memory usage.
- For the knowledge fusion module, co-association matrix fusion yielded consistently high classifier performance; albeit when combined with kernels (as done by DFS-KC) rather than when combined with embeddings (reflected by the poor performance of DFS-EC). However, further exploration of how each representation strategy interplays with each fusion strategy is required to understand this aspect better, which was out of the scope of the current work.
- One of our multimodal data fusion methods (DFS-EC) demonstrated consistently poor classifier performance across all 3 applications. While this method has demonstrated significant success in previous work [5], its poor performance in the current work could be attributed to (a) inability to handle sparse feature spaces (as seen in Dataset *S*_3_), and (b) use of a linear embedding method (PCA) which is likely unable to handle representation of potentially non-linear biomedical data [30].
- Our experimental datasets demonstrated wide variability in terms of the classifier performance associated with the individual data modalities, which had significant bearing on the performance of different multimodal data fusion methods. For example, in dataset *S*_1_ where both modalities showed a relatively high classifier AUC individually, a simple combination of decision representations offered the highest performance amongst the integrated representations (DFS-DD). However, in dataset *S*_2_ where both modalities showed relatively poor discriminability individually, most of the data fusion methods failed to create accurate, discriminatory representations.
- Dataset *S*_2_ was an example of a Big-*P*-Small-*N* (number of features *P*>> number of samples *N*) problem where the large noisy feature space ensured that most representation strategies failed to yield an accurate classifier. In additional experiments involving feature selection (not shown) to assuage this mismatch, we found that kernel-based approaches performed better in the absence of feature selection (i.e. when provided the entire feature space). By contrast, with feature selection applied, LDR-based approaches improved in performance, likely because they could better identify a discriminatory projection for the data.
- Dataset *S*_3_ was an example of a Small-*P*-Big-*N* (number of samples *N*>> number of features *P*) problem, wherein very sparse feature space caused embedding-based methods (DFS-EC, DFS-ES) to throw a number of errors during our experiments. The issue of very few number of input dimensions was further exacerbated by having a large number of samples causing these methods to become more computationally expensive than when *P*>>*N*.
- While one would expect multimodal data fusion strategies to *always* perform better than at least the weaker modality under consideration, our experimental results suggest otherwise. When suboptimal representation or fusion strategies are utilized e.g. using PCA within DFS-EC for representation, or simple structural fusion within DFS-EC, such data fusion methods tend to perform comparably or worse than the individual modalities. Conversely, when a method leverages different modules in a complementary manner (e.g. kernels, weighting, and semi-supervised learning in DFS-KC), we can construct a truly robust, accurate multimodal data fusion predictor.

The most significant finding of our methodological review and experimental results was the variety of factors affect the process of DR-based data fusion. For example, when combining fusion and representation strategies, one should consider how noisy the individual modalities are or how many samples are available for training the predictive model. Thus, while our initial results indicate that kernel-based methods (DFS-KC) yield highly discriminatory predictive models within all 3 biomedical datasets (each of which comprised different heterogenous modalities), a more wide-ranging evaluation is required to ratify this finding. Our current findings do echo previous work demonstrating the high performance offered by kernel-based representations [14,24–26].

**Table 5:** Description of 327 imaging and 146 proteomic features in Dataset *S*_1_ for classifying AD patients from normal controls

T1w MRI	#	Description
FreeSurfer ROIs extracted	327	Subcortical, cortical volumes, surface area, thickness average and standard deviation for Pallidum, Paracentral, Parahippocampal, Opercularis, Pars Orbitalis, Triangularis, Pericalcarine, Cingulate, Frontal, Pareital, Temporal, Caudate, Insula, Occipital etc.
Proteomic data		Description
Plasma proteomics	146	Microglobulin, Macroglobulin, Apolipoproteins, Epidermal growth factors, Immunoglobulins, Interleukins, Insulin, Monocyte Chemotactic Proteins, Macrophage Inflammatory Proteins, Matrix Metalloproteinases etc.

We also acknowledge several additional limitations exist in our study. While we have attempted to diversify in terms of our choice of datasets and methods evaluated in the current study, we did not attempt a comprehensive evaluation of all possible imaging and non-imaging fusion methods. We have instead opted to systematically compare and relate a few representative DR-based multi-modal data fusion strategies in the context of different clinical applications, to provide an overview of the interplay between different individual modules that can comprise a data fusion method. For example our experiments did not explicitly include an examplar of CCA-based methods [61]. Methods we did implement and compare involved directly projecting data either linearly or non-linearly into a reduced embedding space. As CCA based methods optimize for correlations between modalities when projecting them, they did not fit within the strict definition we utilized in this study. Further, none of the datasets considered in this work comprise more than 2 modalities, nor did we examine multi-class or multi-task learning problems. However, our methodological description (as well as the methods we compared in this work), have been described to be easily extensible to multiple data modalities or labels.

Recently, several papers have examined the use of imputation between heterogeneous data modalities i.e. predicting “missing” values on one modality based on available values on a complementary data modality [62–64]. We have instead examined how to combine the information from across heterogeneous modalities to build predictive models. Our framework also specifically focuses on the steps associated with data fusion, rather than the entire pipeline for building predictive models. For example, while we did perform additional experiments regarding the effect of feature selection (not shown), we did not evaluate this in more detail due to the complexity it would add to our experimental design. The effect on the data fusion method of varying the input feature space or the number of samples required for training are also avenues for future work.

## Conclusions

In this paper, we have presented common concepts, methodological choices, and a unifying workflow to address the major challenges in quantitative, heterogeneous multi-modal data integration. In addition to a wide variety of choices for representation and fusion techniques, we have acknowledged the contribution of resampling or weighting approaches; all of which enable the construction of a variety of different data integration approaches which can be tuned for a particular application, dataset, or domain. In addition to providing an overview of different modules, we experimentally implemented and compared 4 representative data fusion methods in the context of 3 clinically significant applications: (a) integrating T2w MRI with spectroscopy for prostate cancer (CaP) diagnosis in vivo, (b) integrating quantitative histomorphometric features with protein expression features (obtained via mass spectrometry) for predicting 5 year biochemical recurrence in CaP patients following radical prostatectomy, and (c) integrating T1w MR imaging with plasma proteomics to discriminate between patients with and without Alzheimers’ Disease.

Our preliminary results indicate that kernel-based representations are highly effective for heterogeneous data fusion problems such as those considered in this work, as seen by the fact that a weighted multi-kernel data fusion method yielded the highest area under the ROC curve of over 0.8 in all three applications considered. Our results also suggest that in situations where the individual modalities demonstrate relatively poor discriminability, many of the data fusion methods may not yield accurate, discriminatory representations either. This implies that when developing such multimodal data fusion schemes, one must account for how noisy or sparse individual modality feature spaces are, as this could significantly affect embedding-based representations. Optimally weighting individual modalities or samples as implemented in the most successful data fusion strategy also appear to have a significant effect on the discriminability of the final integrated representation.

With the increasing relevance of fused diagnostics in personalized healthcare, it is expected that such heterogeneous fusion methods will play an important role in developing more comprehensive predictors for disease diagnosis and outcome.

## Appendix

**Table 6:** Description of 189 histomorphometric and 650 proteomic features in Dataset *S*_2_ to be used to identify patients who will and who will not suffer CaP recurrence within 5 years

Morphological	#	Description
Gland Morphology	100	Area Ratio, distance Ratio, Standard Deviation of Distance, Variance of Distance, Distance Ratio,Perimeter Ratio, Smoothness, Invariant Moment 1–7, Fractal Dimension, Fourier Descriptor 1–10 (Mean, Std. Dev, Median, Min/ Max of each)
Architectural		Description
Voronoi Diagram	12	Polygon area, perimeter, chord length: mean, std. dev., min/max ratio, disorder
Delaunay Triangulation	8	Triangle side length, area: mean, std. dev., min/max ratio, disorder
Minimum Spanning Tree	4	Edge length: mean, std. dev., min/max ratio, disorder
Co-occurring Gland Tensors	39	Entropy, energy: mean, std. dev., range
Gland Subgraphs	26	Eccentricity, Clustering coefficient C, D, and E, largest connected component: mean, std. dev.
Proteomic		Description
Proteins Identified	650	Protein-disulfide isomerase A6, T-complex protein subunit delta, ADP-ribosylation factor 1/3, Protein di-sulfide-isomerase, Ras GTPase-activating-like protein IQGAP2, T-complex protein subunit beta, Ras-related protein Rab-5C, ATP-dependent RNA helicase DX3X/DDX3Y, 40S ribosomal protein S17, Serine/arginine-rich splicing factor 7, Tubulin alpha-1A chain/alpha-3C/D chain/ alpha-3E chain, Laminin subunit alpha-4, Collagen alpha-1 (VIII) chain, Tubulin-tyrosine ligase-like protein 12

**Table 7:** Description of 58 texture and 6 metabolic features in Dataset *S*_3_, extracted from 1.5 Tesla T2w MRI and MRS for identifying prostate cancer (CaP) on a per-voxel basis

Texture features	#	Description
Kirsh Filters	4	X-direction, Y-direction, XY-diagonal, YX-diagonal
Sobel Filters	4	X-direction, Y-direction, XY-diagonal, YX-diagonal
Directional Filters	5	x-Gradient, y-Gradient, Magnitude of Gradient, 2 Diagonal Gradients
First order Gray Level	8	Mean, Median, Standard deviation, Range for window size = 3×3,5×5
Haralick features	13	Contrast Energy, Contrast Inverse Moment, Contrast Average, Contrast Variance, Contrast Entropy, Intensity Average for window size = 3×3, Intensity Variance, Intensity Entropy, Entropy, Energy, Correlation, info. Measure of Correlation 1, Info. Measure of Correlation 2
Gabor filters	24	Filterbank constructed for different combinations of scale and orientation
Metabolic features		Description
Metabolites Identified	6	Area under peaks for choline (*A*_*ch*_), creatine (*A*_*cr*_), citrate (*A*_*cit*_), and ratios (*A*_*ch*_/*A*_*cr*_,*A*_*ch*_/*A*_*cit*_,*A*_*ch*+*cr*_/*A*_*cit*_)
